# Contributing and Terminating Factors of a Large RSV Outbreak in an Adult Hematology and Transplant Unit

**DOI:** 10.1371/currents.outbreaks.3bc85b2a508d205ecc4a5534ecb1f9be

**Published:** 2014-09-19

**Authors:** RSV Outbreak Investigation Team, Elisabeth Aichinger, Paul Schnitzler, Klaus Heeg, Wolfgang Dederers, Michael A. Benz, Silke Buda, Peter Dreger, Gerlinde Egerer, Christoph Eisenbach, Steffen Gei, Walter Haas, Anthony D. Ho, Nicola Lehners, Kai Neben, Guenter Pfaff, Christiane Prifert, Rainer Schwertz, Markus Thalheimer, Christiane Wagner-Wiening, Benedikt Weißbrich, Udo Buchholz

## Abstract

**BACKGROUND:**

In January 2012, an increase of respiratory syncytial virus (RSV) infections on an adult hematology and transplant unit in a German university hospital was detected. We investigated the outbreak to assess its timing and extent and to identify risk factors for transmission.

**METHODS:**

We tested and typed patient samples pro- and retrospectively for RSV. We conducted a cohort and a case-control study. A confirmed outbreak case had laboratory-diagnosed, nosocomially-acquired RSV infection. Possible outbreak cases had pneumonia but were not laboratory-confirmed.

**RESULTS:**

Of 53 outbreak cases, 36 (68%) were confirmed and 17 (32%) possible. Retrospective testing and chart review dated the beginning of the outbreak to November 2011. Patients with community-acquired RSV infection were identified when the community epidemic began in January 2012. In multivariable analysis (controlling for contact with medical personnel, hygiene behaviour and age) patients with active social behaviour were more at risk for RSV infection (odds ratio 23.8, 95% confidence interval, 1.3 to 434.9; p-value, 0.03). Confirmed outbreak cases were more likely than controls to have been accomodated together with a confirmed or possible case before their onset of illness (OR 9.3, 95%CI: 2.1-85.1; p<0.001). Control measures, including isolation of every patient in the unit, initiated until the end of January terminated the outbreak.

**CONCLUSIONS:**

Epidemiological investigations revealed co-accomodation with a case-patient and active social behaviour as likely risk factors for RSV infection. Awareness of and vigorous testing for respiratory viruses in immunosuppressed hospitalised patients is necessary to timely detect cases with outbreak potential. Isolation of patients with respiratory infectious illnesses is crucial to prevent the continuation or occurrence of outbreaks.

## Notice of Corrections

27 Feb 2017: PLOS Currents - Correction: RSV Outbreak Investigation Team. Contributing and Terminating Factors of a Large RSV Outbreak in an Adult Hematology and Transplant Unit. PLOS Currents Outbreaks. 2014 Sep 19. Edition 1. doi: 10.1371/currents.outbreaks.3bc85b2a508d205ecc4a5534ecb1f9be. View Correction.

## Introduction

Infections with respiratory syncytial virus (RSV) are a common cause of usually mild respiratory illness in all age groups, but especially in infants and toddlers^1^. Infants, immunosuppressed adults and elderly persons with underlying chronic conditions are at increased risk for a severe course of disease^2^^,^ 3 ^,^ 4. Among hemato-oncological patients, RSV infection may lead to prolonged viral shedding, longer hospitalisation time and higher case fatality^5^^,^ 6 ^,^ 7 ^,^ 8. Case fatality in this patient group has been reported to range from 0%^9^, 12.5%^10^ to 29%^11^.

Several outbreaks of RSV on adult hemato-oncological wards are described in the literature^7^^,^ 9 ^,^ 11 ^,^ 12. However, evidence is lacking on risk factors for transmission or mortality in RSV patients compared to a non infected control group. Several control measures, such as patient isolation, improved hand hygiene, the use of personal protective equipment, as well as regular screening of patients and staff were reported to have aided in the control of RSV outbreaks^7^^,^ 9 ^,^ 11 ^,^ 12. However, their contribution in stopping or preventing outbreaks has been difficult to document.

In January 2012, the hematology and transplant unit of the University Hospital of Heidelberg, Germany, informed the local health authority about an increase of RSV infections in patients since the beginning of the year. An outbreak investigation was initiated in order to describe (a) the frequency of RSV in the unit before the outbreak, (b) the epidemiology and timing of the outbreak, (c) to investigate RSV introduction onto the unit, (d) risk factors for spread as well as (e) the effect of control measures implemented.

## Materials and Methods

***Setting***

The unit consists of two wards with 20 double occupancy rooms each (ward A and B) and, one floor below, one transplant unit with eight single bed rooms protected through a high efficiency particulate air filtered ventilation system and one high dependency unit with 14 beds. A fitness room for patient use was located on the same floor as - but outside of - wards A and B. Routine hygiene measures to be followed were stipulated in the hospital´s hygiene plan. This included the use of handrub solution from dispensers placed in front of every patient room and the use of personal protective equipment (gowns, masks and gloves) when entering the room of severely immunocomprimised patients.

***Outbreak management***

After the first RSV cases occured in early January, hygiene measures such as the use of disposable gowns, masks and gloves and thorough hand hygiene were reinforced. Infected patients were isolated in single rooms or cohort isolated with another RSV infected patient. All in-patients and newly admitted patients were screened for RSV. In late January 2012, all patients were isolated, independently of their RSV status, and elective admissions were suspended. Health care workers (HCW) were cohorted according to patient RSV status. Visitors and HCW with respiratory symptoms were banned from entering the wards or suspended from work until a negative test result for RSV was obtained, respectively.

In the absence of nosocomial transmissions for a period of four weeks, the clinic management officially declared the end of the outbreak on February 27^th^.

***Epidemiological methods***

We reviewed documentation on laboratory RSV tests from 2008 until 2011 of patients hospitalised in the unit. We screened medical charts and patient location records from all patients hospitalised for at least three days on one of the four wards of the unit between November 01, 2011 and end of January 2012. We collected information on demographics, date of admission, respiratory symptoms and date of the first positive RSV test. A “respiratory episode” fulfilled one of the following criteria: (i) documented respiratory symptoms with fever, (ii) documented upper respiratory tract diagnoses, such as bronchitis or rhinitis, or (iii) pneumonia diagnosed through computer tomography (CT) or clinically. Because the incubation period for RSV can vary from two to eight days^13^^,^ 14 ^,^ 15, we recorded a respiratory episode if the patient was hospitalised on one of the wards 2-8 days before the onset of symptoms. We defined the first day of a respiratory episode as the onset of respiratory symptoms or the date when pneumonia was diagnosed. For patients with laboratory-confirmed RSV infection without a respiratory episode we recorded the date of the RSV diagnosis as the beginning of the respiratory episode. Case definitions used are listed in Figure 1.

**Case definitions used during the outbreak investigation, Heidelberg, November 2011- February 2012.**
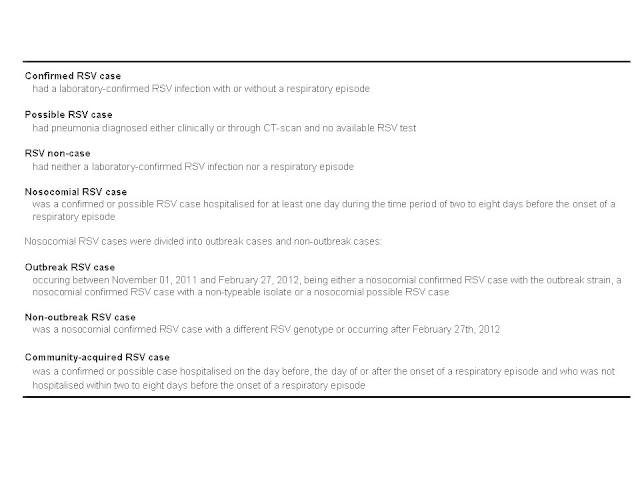

For outbreak cases we defined the ward where the infection was most likely acquired as the ward on which the patient was accomodated four days prior to onset of the respiratory episode.

To investigate possible risk factors contributing to the transmission of RSV on the wards we performed two analytical studies. A cohort study aimed to identify risk and preventive factors for the transmission of RSV, such as contact behaviour and hygiene practices of patients (Figure 2). We asked patients about the frequency of leaving their room or the ward, frequency of using the fitness room, number of other in-patients known by name, frequency of conversing with other patients and self-assessment of sociableness. Hygiene practices were elicited through questions about the frequency of handwashing in general, handwashing before meals and if the patient tried to avoid touching his/her own face during the day.

**List of variables used in the cohort study:**
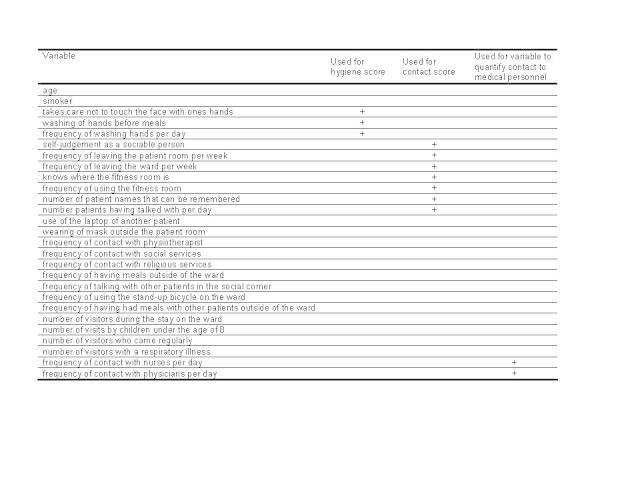

The time period of interest was January 01 to 12, 2012, i.e. before regular screening of all patients and rigorous control measures had begun. The cohort consisted of all patients who were hospitalised on the two main wards in this time period. We passed a paper questionnaire to in-patients and sent the questionnaire by mail to patients who were already discharged. Questionnaires were entered into EpiData (EpiData Association, Odense, Denmark) and analysed with STATA12 software (College Station, TX, USA). Numeric variables were analysed with Kruskal-Wallis equality-of-populations rank test and categorical variables with Fisher´s exact Chi-Square test. We considered two-sided p-values of less than 0.05 as statistically significant. We created a “contact score” of the six relevant variables (one being dependent on a seventh) with a maximum of 6 points. To do this we imputed for the few missing entries the median of the entries of the other participants. Contact score was dichotomised with a cut-off at 3 points. Similarly, a hygiene score integrated results of the three hygiene variables. Thus, a maximum of 3 points could be reached, imputation was done the same way as for the contact score. Hygiene score was also dichotomised with a cut-off at 2 points. To analyse the degree of contact to health care personnel we first imputed few missing values (based on the median of the fields with information), then added the daily frequency of contact to nurses and physicians, and finally created a binary variable with a cut-off at the median of the values. Except for the variables coding for the contact with health care workers (where entries were missing more frequently among female participants) imputed variables were either missing only in single questionnaires or missing values were not distributed differently by sex or age. For all variables we conducted univariable analysis. Moreover we performed a multivariable analysis where we included statistically significant variables and other variables deemed relevant, i.e. the two scores as well as the variable coding for the degree of contact to health care personnel, as well as age.

We conducted a matched case-control study to evaluate whether a laboratory-confirmed outbreak case was more likely to have shared a double room with an infectious confirmed or possible outbreak case in the time period of two to eight days before the onset of symptoms. We considered a (confirmed or possible) RSV case to be infectious from the first day of his or her respiratory episode. Our control group were RSV non-cases who were matched according to their simultaneous stay on the unit during the time of onset of the respiratory episode of the outbreak case and the eight days before. We attempted to match two non-cases to one case. We conducted univariable analysis of all variables as well as a multivariable analysis of the co-accomodation variable controlling for age as additional explanatory variable. A second goal of the case-control study was to assess if the chance of dying while being hospitalised was different for confirmed outbreak cases and controls. We reviewed if confirmed outbreak cases had died within the course of their RSV infection and for the controls if they had died while being hospitalised after the onset date of the respiratory episode of the matched case. Underlying diseases were not assessed. We calculated matched odds ratios (OR) with 95% confidence intervals using exact logistic regression controlling for age.

***Ethics statement***

The study was approved by the institutional review board of the hospital. Patients willing to participate signed a consent form that was sent to us together with the filled questionnaire.

***Virological methods***

To assess the timing of the RSV wave in the community we collected RSV data from the pediatric unit of the hospital where children with respiratory symptoms were tested for RSV on admission. To identify further cases on the hemato-oncological wards, from January 13^th^, 2012, all unit patients were tested for RSV using polymerase chain reaction (PCR) twice a week as well as HCW when being symptomatic. Stored laboratory samples of hemato-oncological patients who suffered from respiratory symptoms in November and December 2011 were retrospectively tested for RSV. All available RSV positive isolates of hemato-oncological patients were sent to the Consultant Laboratory in Wuerzburg for RSV for genotyping. Environmental swabs taken in the room of a confirmed case were tested for RSV.

To assess the frequency of the typed outbreak strain among other patients in Germany, isolates from other public health and private laboratories collected during the winter season of 2011/2012 throughout the country were sent to the Consultant Laboratory for RSV.

## Results

***Retrospective analysis of laboratory data for RSV***

Between January 2008 and November 2011 few samples from patients of the four hematology and transplant wards were tested for RSV: in 2008 none, and from 2009 to November 2011 a monthly maximum of four (Figure 3). In these four years, excluding December 2011, only one patient sample tested positive for RSV.

**Number of positive (blue) and negative (red) RSV tests in specimens of hemato-oncological patients; Heidelberg, 2008-2011.**
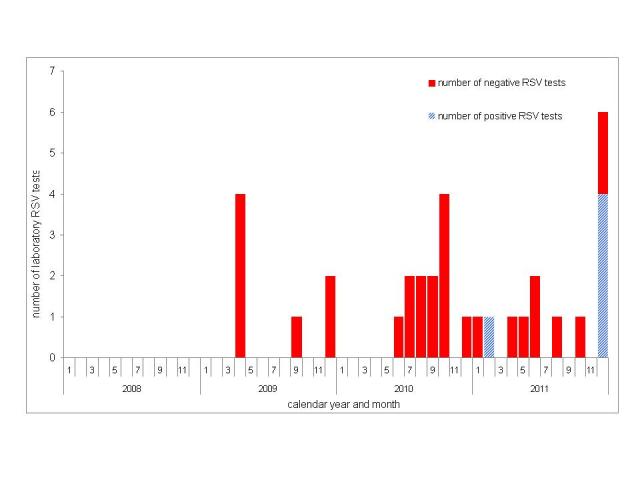

***Descriptive epidemiology of the outbreak***

A total of 56 nosocomial RSV cases were identified. Three nosocomial RSV patients were categorised as non-outbreak cases, leaving a total of 53 outbreak cases of which 36 (68%) ****were confirmed and 17 (32%) possible (Figure 4). In addition, we identified 24 community-acquired RSV cases, of whom 19 (79%) were laboratory-confirmed and 5 (21%) possible.

**Number (blue bars; left y-axis) and incidence rate (red line; right y-axis) of RSV outbreak cases on a hemato-oncological unit by week of onset or (in asymptomatic cases) by week of first detection (36 laboratory-confirmed, 17 possible cases); Heidelberg, November 2011 to February 2012.**
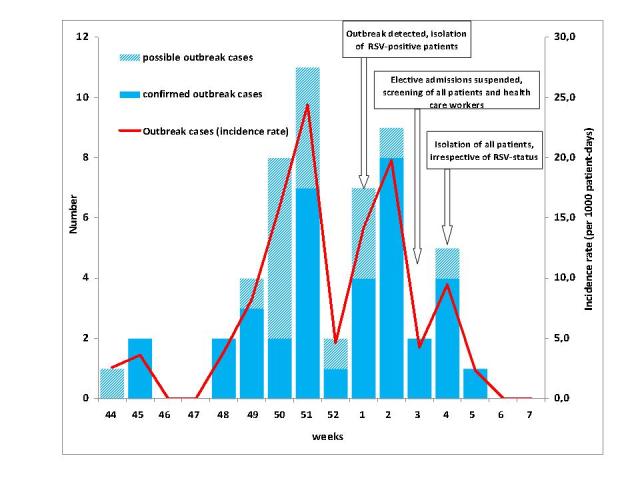

The onset of respiratory episodes of outbreak cases dated between November 02, 2011, and January 30, 2012, with a peak in the second half of December and first half of January. After the implementation of hygiene measures, isolation of all patients and the suspension of patient admissions the last outbreak case occurred on January 30, 2012.

Cases with community-acquired RSV infection occurred from late December to March, with most cases detected between January and February 2012 (Figure 5). The curve of community-acquired cases coincided with the curve of RSV infections in the pediatric unit of the university hospital (Figure 5).

**RSV outbreak cases (N=53), community-acquired cases (N=24) and pediatric RSV cases (N=109) by week of onset of the respiratory episode (haemato-oncological patients) or week of admission (pediatric cases), November 2011- April 2012.**
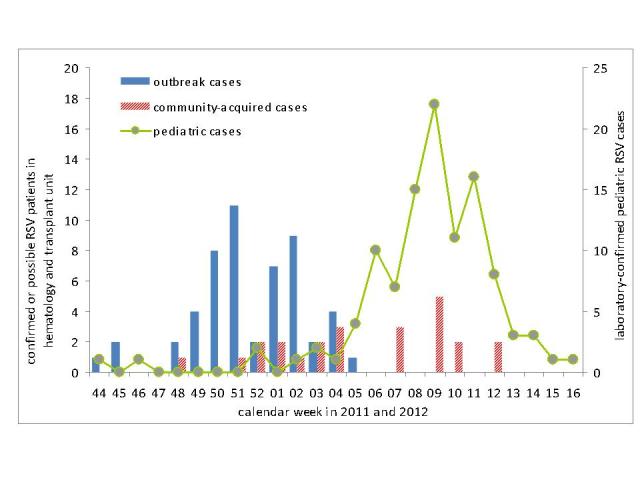

Mean age of the 53 outbreak cases was 58 years (median 59, range 25-78 years) and 53% were male. Age and sex did not differ between confirmed and possible outbreak cases. The average interval for all 53 outbreak cases from hospital admission to the beginning of the respiratory episode was 16 days (median 12, interquartile bounds 9-17, range 1-69 days) and did not differ significantly between the group of confirmed and possible cases (p-value=0.83). 23 (64%) of 36 confirmed cases were diagnosed with pneumonia, six (17%) presented with mild respiratory symptoms and seven (19%) were asymptomatic. By definition, all possible cases were diagnosed with pneumonia. Of the 36 confirmed outbreak cases 11 patients (31%) had died while being hospitalised. Of 17 possible outbreak cases, six (35%) had died while being hospitalised. Controlling for age, confirmed cases were 8.1 times more likely to die while being hospitalised when compared to controls (95%CI=1.8-74.9; p<0.003).

When determining the ward where patients had likely acquired their RSV infection we identified ward A for 36% (19 cases), ward B for 34% (18 cases), the high dependency unit for 19% (10 cases) and the transplant unit for six patients (11%) of all 53 outbreak cases (Figure 6). For a period of seven weeks, RSV infections had occurred on all four wards of the unit.

**RSV outbreak cases (N=53) by ward of possible acquisition of infection. Depicted is the ward on which patients were accomodated four days prior to onset of their respiratory episode; Heidelberg, November 2011- February 2012.**
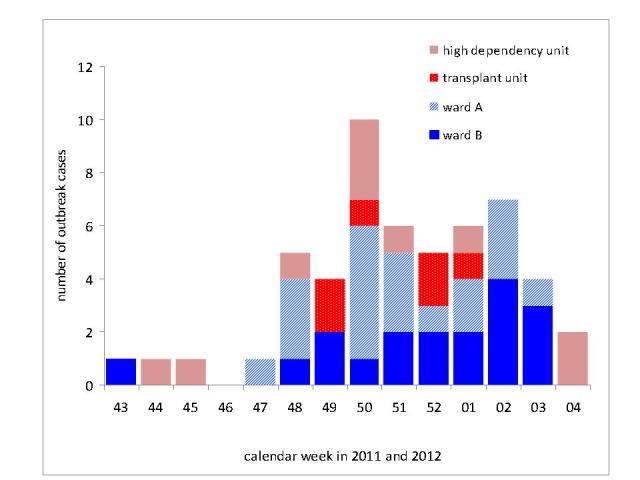

***Virological results*****

The Consultant Laboratory genotyped isolates of confirmed nosocomial RSV cases. 34 (87%) of 39 could not be distinguished from each other and belonged to group A, genotype GA2 (outbreak strain)^16^. Two had a different sequence. Three could not be genotyped due to technical reasons. Among 12 typeable isolates from community-acquired cases, eight (67%) were indistinguishable from the outbreak strain. Genotyping of isolates collected throughout Germany revealed the outbreak strain in 14 (5%) of 291 patients. Testing of symptomatic HCW in late January identified nine individuals infected with RSV. However, none of the specimens of HCW could be genotyped as the viral load among all HCW was below the threshold necessary for genotyping. Of five environmental swabs taken, the specimens from the door handle and the sink were positive for RSV.

***Serial testing of RSV patients***

Half of RSV positive patients shed viral RNA for 3-4 weeks^16^. Among 44 patients with at least one sample obtained after the onset of the respiratory episode, or – in asymptomatic patients – after the first positive test there were 9 (20%) patients who tested positive again after two consecutive negative tests.

***Cohort study***

A total of 35 (59% of 59) questionnaires were returned, of which 34 were correctly filled in and could be analysed. Non-responders were not significantly different from responders regarding age, gender or duration of hospital stay. Patients participating in the study were 44% male and the median age was 56 years. In univariable analysis patients who had a more sociable behaviour (measured by the contact score) were more likely to become a case, all other variables were not statistically significant (Figure 7). In multivariable analysis we included contact score, hygiene score and age (in tertiles). When controlling for these variables the association of active sociable behaviour and becoming a case remained. Practicing a more hygienic behaviour was negatively associated with becoming a case, but results were not statistically significant.

**Uni- and multivariable analysis of contact behaviour, hygienic behaviour, contact with health care personnel, age and sex:**
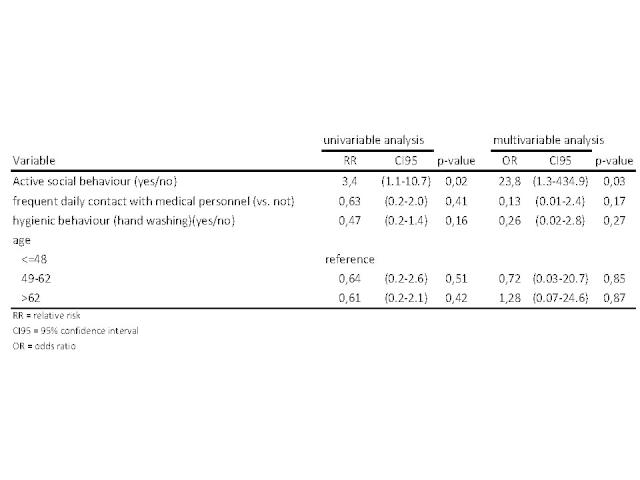

***Case-Control study***

We included 35 laboratory-confirmed outbreak cases and 61 controls. Both groups did not differ significantly in age or gender. In univariable analysis only co-accomation with a potentially infectious possible or confirmed case was significant (Figure 8). Confirmed cases were more likely to have been accommodated together with a confirmed or possible case before their onset of illness (Figure 8). Controlling for age did not change the association ((OR 9.3; 95%CI, 2.1 to 85.1; p-value, <0.001)). Fifteen (43%) of 35 cases might be explained through this mechanism.

**Uni- and multivariable analysis of co-accomodation of cases/non-cases with potentially infectious cases/noncases as well as age.**
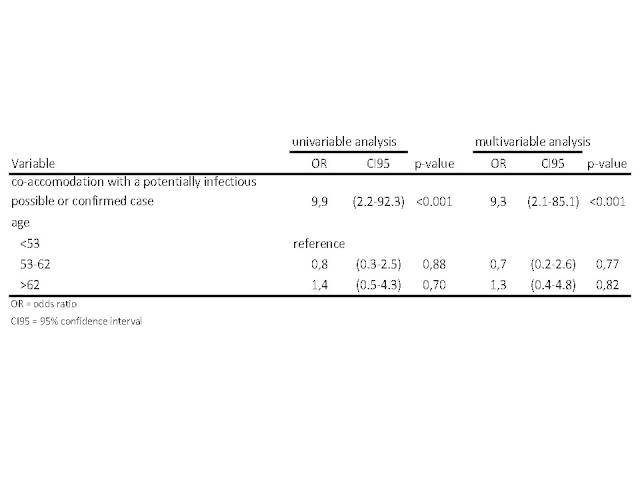

## Discussion

We investigated a large, nosocomial RSV outbreak that began two months before the outbreak was recognized. Transmission had likely occurred on all four wards and risk factors for spread included sharing a room with a potentially infectious confirmed or possible case-patient. The vast majority of RSV isolates of confirmed nosocomial cases was indistinguishable from each other. Community-acquired cases identified among in-patients only occurred when the community RSV epidemic began. Viral shedding in this patient group occured for several weeks and a substantial number of patients tested positive again after two negative RSV test results.

In the years 2008-2011 only a small number of hemato-oncological patients were tested for infection with RSV. Generally, when searched for systematically, RSV can be diagnosed frequently. A three-year survey of adult cancer patients in Houston, Texas, identified a respiratory virus in one third of patients with a respiratory illness episode, and one third of these were RSV^17^. Thus, in this setting it is likely that a number of single cases or perhaps even outbreaks of RSV infections could have been missed in the past.

Reasons for the large outbreak size include not only the size of the unit and the late recognition, but also the fact that the turnover of patients has accelerated substantially in the last 10-15 years. In immunocompromised patients, respiratory viral infections may be clinically indistinguishable from those caused by opportunistic pathogens commonly associated with these type of patients^5^ and respiratory infections may be mimicked by other – disease or treatment related – conditions. It is therefore prudent in this vulnerable population to set a very low threshold to test for RSV and other viral and bacterial pathogens with outbreak potential. Because young children aged less than five years share the susceptibility to many pathogens, such as influenza or human metapneumovirus, with hemato-oncological patients, information from a local surveillance system and/or a close link with the pediatric department may help to increase awareness towards specific pathogens. To be better prepared for future outbreak threats and in keeping with guidelines recommended by the European Conference on Infections in Leukaemia^18^, the hospital laboratory has established diagnostics for a broad spectrum of pathogens with outbreak potential.

Case-fatality of RSV among these patients reported in the literature has varied widely and ranged from 0%^9^ and 12.5%^10^ to 29%^11^. While the contribution of RSV to a fatal course in an individual patient may be difficult to assess, comparison of case fatality with non-cases revealed that confirmed outbreak cases were significantly more likely to die.

Regarding the question if there was a single or multiple introduction of the virus onto the wards, all of the following facts strongly support the scenario that the virus was introduced once and then spread nosocomially: (1) most nosocomial confirmed RSV cases revealed an identical genotype; (2) the community epidemic of RSV had not started yet when the outbreak occurred, so at this time the incidence of RSV in the community was very low, thus, multiple introduction from the community highly unlikely; (3) although the outbreak strain was detected throughout Germany, it was identified in low proportions.

To our knowledge, there are no studies so far that have analytically investigated risk factors for transmission of RSV in nosocomial outbreaks on adult hemato-oncological units. This may be partly due to the fact that most reported outbreaks consisted of less than ten cases^7^^,^ 9 ^,^ 19, thereby limiting the statistical power to conduct epidemiological studies. In this outbreak we have found that cases were more likely than controls to have shared a double-room with another potentially infectious patient. This mechanism would explain 43% of laboratory-confirmed cases. In addition, other factors such as socializing behaviour of patients may also have facilitated transmission. A possible contribution of health care workers was assessed by the frequency of daily contact and did not show an association. The potential contribution of HCW could not be assessed more formally due to complex contact patterns of HCW, but transmission also on the high dependency unit where patients were isolated in single rooms suggests that also HCW may have contributed to transmission. Hall et al. argue that infections in otherwise immunocompetent persons “lack distinctive signs and initially sometimes lack severity” (with 15% of infections being asymptomatic), that RSV infections among HCW “may not be recognized, and staff may become effective but occult vectors for transmission on the ward”^5^. The identification of RSV infections in nine HCW who - under the pre-outbreak regime would have gone undetected - is a case in point. The fact that the virus was found in environmental samples and that there was a (statistically non-significant) indication that persons with higher hygiene standards were less likely to develop laboratory-confirmed RSV infection suggests that also fomite transmission, i.e. touching surfaces with consequent self-inoculation, played a (perhaps minor) role.

Similar to a regimen successfully applied by Lavergne in Montreal^10^, outbreak management (as implemented in Januar 2012) included cohorting of RSV patients, accommodation of all patients in single rooms as well as testing and suspending of HCW with respiratory infections. These measures did not only stop the ongoing outbreak with the last case identified on January 30. At the same time, due to the intensified patient screening, an increasing number of admitted patients as well as HCW were diagnosed with RSV infections acquired in the community posing a considerable threat of a renewed flare-up of nosocomial RSV infections. The installed measures mentioned above, however, were effective in preventing a second wave of nosocomially-acquired cases. In particular, these findings support international recommendations stating that any HCW or visitor with a respiratory infection should abstain from work or refrain from visiting^18^^,^ 20 ^,^ 21, regardless if a pathogen was detected or not.

RSV shedding among hemato-oncological patients of the unit was shown to be prolonged^16^. However, it also seems to be of variable intensity. Twenty percent of patients tested positive again even after two consecutive negative RSV tests. Although it is unclear if PCR positivity correlates with infectiousness, it may be prudent to assume that these type of patients remain RSV positive for the duration of their hospital stay.

There are several limitations that need to be acknowledged. Some aspects of the descriptive analysis are based on the information included in patient charts which are filled in by attending physicians and were therefore not standardised and could be incomplete. The number of possible cases might have been slightly overestimated given the fact that we only assessed whether or not patients suffered from pneumonia, regardless if another pathogen was already (or later) identified, which, however, was rarely the case. Furthermore, we did not collect any information on the degree of immunosupression, underlying conditions or the treatment administered which might be confounders in the analysis for the risk of death. The use of an incubation period of 2-8 days may have led to misclassification of patients as nosocomial when in fact they were not. However, the potential mistake should have a small effect. When applying an incubation period of four days, only two confirmed and two possible cases would not be categorised as nosocomial anymore. Finally, using a simple imputation method has increased the power of the cohort study to find a difference if there is a difference; we cannot exclude of course the possibility that values were assigned wrongly to participants.

In conclusion, we report about the lessons learnt from this large RSV outbreak on an adult hemato-oncological unit: we have demonstrated the importance to test immunosuppressed symptomatic patients in this population for pathogens that have the potential to cause nosocomial cases or even outbreaks. Co-accomodation with a RSV infected patient appeared to be a risk factor for infection, but also active social behaviour may have played a contributing role. We suggest that RSV positive haemato-oncological patients should be regarded as infectious for the duration of their hospital stay. HCW with mild respiratory infections need to be aware that they, too, may be an infectious source for their susceptible patients. Finally, stringent outbreak management, including isolation of all patients, intensified barrier measures, as well as virological screening of admitted patients and HCW with respiratory illnesses, appear to be very effective in halting or preventing outbreaks.

## *Members of the RSV Outbreak Investigation Team

Elisabeth Aichinger (1,2), Paul Schnitzler (3), Klaus Heeg (3), Wolfgang Dederer (4), Michael A. Benz (3), Silke Buda (5), Peter Dreger (6), Gerlinde Egerer (6), Christoph Eisenbach (6), Steffen Geis (3), Walter Haas (5), Anthony D. Ho (6), Nicola Lehners (6), Kai Neben (6), Guenter Pfaff (1), Christiane Prifert (7), Rainer Schwertz (8), Markus Thalheimer (4), Christiane Wagner-Wiening (1), Benedikt Weißbrich (7), Udo Buchholz (5)

^1^ Baden-Wuerttemberg State Health Office, Stuttgart, Germany

^2^ Postgraduate Training for Applied Epidemiology, Robert Koch Institute, Germany affiliated to the European Programme for Intervention Epidemiology Training, ECDC, Sweden

^3^ Department of Infectious Diseases, University of Heidelberg, Heidelberg, Germany

^4^ Department for Quality Management and Medical Controlling, University of Heidelberg, Germany

^5^ Robert Koch Institute, Berlin, Germany

^6^ Department of Internal Medicine, University of Heidelberg, Heidelberg, Germany

^7^ Institute of Virology and Immunobiology, University of Wuerzburg, Wuerzburg, Germany

^8^ Local Health Authority Rhein-Neckar-Kreis, Heidelberg, Germany
