# Correction: Contributing and Terminating Factors of a Large RSV Outbreak in an Adult Hematology and Transplant Unit

**DOI:** 10.1371/currents.outbreaks.e0604bd4ebdbde1bd88392cc29f63a9c

**Published:** 2017-02-27

**Authors:** PLOS Currents

## Correction

The author's name is incorrect. The correct name is: RSV Nosocomial Outbreak Investigation Team. The correct citation is: RSV Nosocomial Outbreak Investigation Team. Contributing and Terminating Factors of a Large RSV Outbreak in an Adult Hematology and Transplant Unit. PLOS Currents Outbreaks. 2014 Sep 19. Edition 1. doi: 10.1371/currents.outbreaks.3bc85b2a508d205ecc4a5534ecb1f9be.
